# Dietary Patterns and Hepatocellular Carcinoma Risk among US Adults

**DOI:** 10.3390/nu13062011

**Published:** 2021-06-11

**Authors:** Iman Moussa, Rena S. Day, Ruosha Li, Xianglin L. Du, Ahmed O. Kaseb, Prasun K. Jalal, Carrie Daniel-MacDougall, Rikita I. Hatia, Ahmed Abdelhakeem, Asif Rashid, Yun Shin Chun, Donghui Li, Manal M. Hassan

**Affiliations:** 1 Department of Epidemiology, Human Genetics and Environmental Science, School of Public Health, The University of Texas Health Science Center at Houston, Houston, TX 77030, USA; iman.moussa@bcm.edu (I.M.); xianglin.l.du@uth.tmc.edu (X.L.D.); 2 Department of Epidemiology, Human Genetics and Environmental Sciences, Southwest Center for Occupational and Environmental Health, Michael & Susan Dell Center for Healthy Living, School of Public Health, The University of Texas Health Science Center at Houston, Houston, TX 77030, USA; rena.s.day@uth.tmc.edu; 3 Department of Biostatistics and Data Science, School of Public Health, The University of Texas Health Science Center at Houston, Houston, TX 77030, USA; ruosha.li@uth.tmc.edu; 4 Department of Gastrointestinal Medical Oncology, The University of Texas MD Anderson Cancer Center, Houston, TX 77030, USA; akaseb@mdanderson.org (A.O.K.); aaabdelhakeem@mdanderson.org (A.A.); dli@mdanderson.org (D.L.); 5 Division of Gastroenterology and Hepatology, Department of Medicine, Baylor College of Medicine, Houston, TX 77030, USA; jalal@bcm.edu; 6 Department of Epidemiology, The University of Texas MD Anderson Cancer Center, Houston, TX 77030, USA; CDaniel@mdanderson.org (C.D.-M.); Rhatia@mdanderson.org (R.I.H.); 7 Department of Pathology, The University of Texas MD Anderson Cancer Center, Houston, TX 77030, USA; arashid@mdanderson.org; 8 Department of Surgical Oncology, Division of Surgery, The University of Texas MD Anderson Cancer Center, Houston, TX 77030, USA; yschun@mdanderson.org

**Keywords:** liver cancer, dietary patterns, case control

## Abstract

The objective of this study was to assess the association between dietary patterns and risk of hepatocellular carcinoma (HCC) among US adults in a hospital-based case-control study. We analyzed data from 641 cases and 1002 controls recruited at The University of Texas MD Anderson Cancer Center during 2001–2018. Cases were patients with a pathologically or radiologically confirmed new diagnosis of HCC; controls were cancer-free spouses of patients with cancers other than gastrointestinal, lung, liver, or head and neck cancer. Cases and controls were frequency-matched by age and sex. Dietary patterns were identified by principal component analysis. Odds ratios (ORs) and corresponding confidence intervals (CIs) were computed using unconditional logistic regression with adjustment for major HCC risk factors, including hepatitis B virus and hepatitis C virus infection. A vegetable-based dietary pattern was inversely associated with HCC risk (highest compared with lowest tertile: OR 0.66, 95% CI 0.46–0.94). A Western diet pattern was directly associated with HCC risk (highest compared with lowest tertile: OR 1.79, 95% CI 1.19–2.69). These findings emphasize the potential role of dietary intake in HCC prevention and clinical management.

## 1. Introduction

Hepatocellular carcinoma (HCC) is the sixth most common cancer and the third leading cause of cancer-related deaths in the world [1,2]. HCC advances rapidly and is usually diagnosed at late stages. The main etiologic factors of HCC are hepatitis B virus (HBV) and hepatitis C virus (HCV) infections, alcohol consumption, type 2 diabetes mellitus, cigarette smoking, and obesity [3,4,5,6,7]. In Western countries, other risk factors such as metabolic syndrome and non-alcoholic fatty liver disease were shown to be associated with increased incidence of HCC in the past decade [8,9,10,11]. Given the role of diet in the development of obesity, metabolic syndrome, and steatosis, it is important to study the effect of diet on HCC risk, especially given that diet is a modifiable lifestyle factor.

Dietary pattern analysis allows assessment of the effect of a combination of foods, instead of a single nutrient or food item, on disease risk. Dietary patterns are reflective of the eating behavior of an overall population because foods and nutrients are not consumed in isolation. In addition, dietary pattern analysis results can be used to generate public health interventions to reduce risk of disease. Two studies have assessed the association between dietary patterns and HCC risk within a US population, but these studies did not consider the critical role of HBV and HCV infection in the etiology of HCC [12,13]. The objective of the current study was to identify dietary patterns of study participants (using principal component analysis (PCA)) and to examine the association of these dietary patterns with HCC while considering the effect of major etiologic factors, including HBV and HCV. 

## 2. Materials and Methods

The current investigation is part of an ongoing hospital-based case-control study, approved by the institutional review board at The University Texas MD Anderson Cancer Center. Written informed consent for participation was obtained from each participant. Participants were recruited between 7 March 2001 and 5 March 2018. New patients with HCC were prospectively enrolled at the MD Anderson gastrointestinal medical oncology and surgical oncology outpatient clinics. Cases included patients with a pathologically or radiologically confirmed diagnosis of HCC following clinical guidelines by the American Association for the Study of Liver Disease (AASLD) [14]. Controls were cancer-free and genetically unrelated family members (as spouses) of patients with cancers other than gastrointestinal, lung, liver, or head and neck cancer. Controls were recruited from MD Anderson central diagnostic radiology clinics. The spouse of an HCC case could not be a control. Cases and controls were US residents and were frequency-matched by age (±5 years) and sex. 

**Figure 1:**
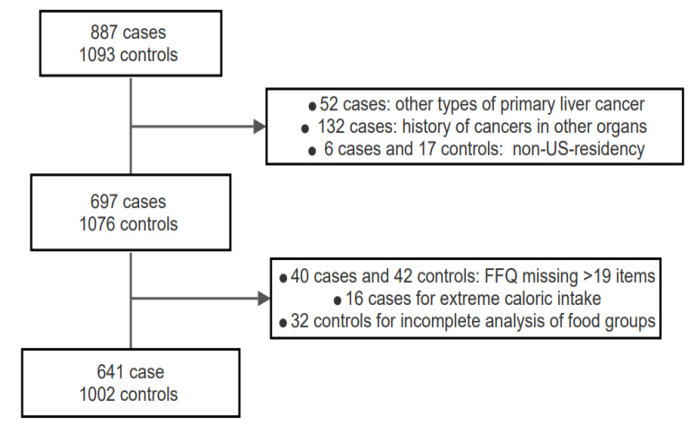
Inclusion flow chart for cases and controls.

We recruited 887 cases and 1093 controls, and our analysis included those who completed a food frequency questionnaire (FFQ). We excluded 52 cases for having other types of primary liver cancer, such as cholangiocarcinoma and fibrolamellar HCC. We excluded 132 cases with a prior history of cancers in other organs (67 cases with skin cancer were not excluded). An additional 6 cases and 17 controls were excluded for non-US-residency. We excluded 40 cases and 42 controls for missing >19 items on the FFQ, 16 cases for extreme caloric intake (5 cases for caloric intake > 6000 kcal and 11 cases for caloric intake < 500 kcal), and 32 controls for incomplete analysis of food groups. After these exclusions, 641 cases and 1002 controls remained for analysis (Figure 1). Detailed information on the assessment and definition of risk factors was described previously [15]. At recruitment, cases and controls were interviewed in person using a validated structured questionnaire to collect information on sociodemographic factors, including education, race, cigarette smoking, alcohol consumption, history of type 2 diabetes mellitus, medical history, family history of cancer, and height and weight history [16]. Participants’ weight history was not collected from the beginning of the study; hence a total of 464 participants (134 cases and 330 controls) were missing body mass index (BMI) data.

Blood samples were collected from cases and controls and tested for HCV antibodies, hepatitis B surface antigen, and antibodies to hepatitis B core antigen. The following clinical variables were abstracted from patients’ medical records: cirrhosis, HCC treatment modalities received, information for disease staging, survival data, pathologic differentiation, vascular invasion, metastasis, lymph node involvement, tumor nodularity, and tumor size. Cirrhosis was determined by pathologic findings (diagnostic biopsies) and computed tomography scans, or by documented clinical signs of cirrhosis such as ascites, bleeding esophageal varices and hepatic encephalopathy. The Willett semi-quantitative FFQ [17] was used to assess the usual dietary intake of participants during the past year (the year prior to cancer diagnosis for cases and prior to recruitment for controls). The FFQ included the following categories of foods: dietary supplements, dairy foods, fruits, vegetables, eggs and meat, breads and cereals, beverages, and sweets. The FFQ included standard portion sizes and frequency of consumption options ranging from “never, or less than once per month” to “≥6 per day” during the past year. Completed FFQs were processed by the Department of Nutrition at the Harvard T. H. Chan School of Public Health.

We used PCA to determine dietary patterns. The FFQ food items were reduced to 35 food groups (Table 1) based on nutrient profiles as suggested by other studies. The PROC FACTOR procedure in SAS version 9.4 (SAS Institute, Cary, NC, USA) was used to perform PCA. PCA was done with orthogonal varimax rotation and factors from PCA were retained by evaluating their eigenvalues, scree plots, and interpretability. From this analysis, two factors were extracted (eigen value >2.5) and named based on the food groups with the highest loading (loading >0.2; Table 2). Factor scores for each participant, for each of the two factors, were computed by multiplying the food group factor loading by the participant’s intake of the respective food group summed across all food groups [18,19]. Tertiles of factor scores were based on the distribution of factor scores among the controls with the lowest tertile as the reference. The trends of demographic and lifestyle characteristics across tertiles of dietary patterns were estimated by the Cochran–Armitage trend and Cochran–Mantel–Haenszel test. The Jonckheere–Terpstra test was used to assess linear trends of nutrient and food intake across dietary pattern tertiles.

We used multivariable unconditional logistic regression analysis to compute adjusted odds ratios (OR) and 95% confidence intervals (CI) of the highest tertile of factor scores compared with the lowest tertile of factor scores. Selection of other variables for adjustment in the regression models was based on the change in estimate approach [20]. A covariate causing a 10% change in the estimated OR for the dietary pattern was considered a confounder and included in the final model. We evaluated the following risk factors for confounding: cigarette smoking (no smoking, moderate smoking (≤20 pack-years of smoking), or heavy smoking (>20 pack-years of smoking)), alcohol consumption (no drinking, moderate drinking (≤60 mL of ethanol per day), or heavy drinking (>60 mL of ethanol per day)), education level (less than a college education or college education or higher), race (non-Hispanic White or other race (Hispanic, African American, and Asian)), family history of cancer (yes or no), history of type 2 diabetes mellitus (no diabetes, diabetes for ≤1 year of HCC diagnosis, or diabetes for >1 year of HCC diagnosis), average BMI during early adulthood (mid-20s to mid-40s, with BMI categorized as normal weight (≤24.9 kg/m^2^), overweight (25–29.9 kg/m^2^), or obese (≥30 kg/m^2^)), HBV/HCV infection (none or infection with either or both), total calorie intake (tertiles), and multivitamin use (yes or no).

Tests for linear trend were performed by entering tertile scores of the dietary patterns as continuous variables in the models [21]. We stratified regression models by HBV/HCV infection, sex and age to assess for potential effect measure modification of the association between dietary patterns and HCC risk due to these covariates. Given that cirrhosis is a primary risk factor for HCC development and that patients with cirrhosis often change their dietary habits, sensitivity analyses were conducted to evaluate the association between dietary patterns and HCC among non-cirrhotic patients to rule out possible reverse causation. Additionally, we examined the association of dietary patterns with cirrhosis among the cases (cirrhotic compared with non-cirrhotic HCC patients). Potential multiplicative interaction between dietary patterns and HCC risk factors (age, sex, and HBV/HCV infection) was evaluated by including interaction terms formed by the product of the risk factor of interest and tertile of dietary pattern in the logistic regression model. 

All statistical analyses were completed using SAS version 9.4 (SAS Institute Inc., Cary, NC, USA) with 2-sided tests. *p* < 0.05 was considered statistically significant.

## 3. Results

In PCA, we identified two major dietary patterns based on eigenvalues and scree plots (Table 2). We described the patterns as a “vegetable-based” dietary pattern characterized by high intake of many vegetables and a “Western diet” pattern characterized by high factor loading of red meat, processed meat, snacks, and sweets. These two patterns explained 19.9% of the total variance in food intake.

Table 3 shows the distribution of general characteristics of cases and controls. Cases were more likely to be smokers, have type 2 diabetes mellitus, be obese, and consume alcohol compared with controls. Mean age (±SD) was 62.9 ± 10.9 years for cases and 60.0 ± 10.7 years for controls. The male:female ratio was 2.8:1 among cases. About 45% of cases and 2% of controls had evidence of chronic HBV or history of HBV and/or HCV infection.

Overall, the distribution of demographic and lifestyle factors of controls was similar across dietary pattern tertiles, with a few exceptions (Table 4). The proportion of male participants and participants younger than 60 years decreased and the proportion of multivitamin users increased across tertiles of the vegetable-based pattern. By contrast, the proportion of males increased across tertiles of the Western diet pattern. 

Results of the multivariable analyses are shown in Table 5. The vegetable-based pattern was inversely associated with HCC risk. Participants in the third tertile of the vegetable-based pattern had a 34% reduced risk of HCC compared with those in the first tertile. In contrast, a direct association was observed between the Western diet pattern and HCC. Compared with those in the first tertile, participants in the third tertile of the Western diet pattern had a 79% increased risk of HCC. 

We also examined the identified associations between HCC risk and dietary pattern among non-cirrhotic patients. The results were similar to those observed in the entire study population, although in the non-cirrhotic subgroup the associations were not statistically significant for those in the third tertile compared with the first tertile of the vegetable-based pattern (OR 0.70, 95% CI 0.46–1.06); for those in the third tertile compared with the first tertile of the Western diet pattern (OR 1.81, 95% CI 1.14–2.87). No association was found between dietary pattern and risk of cirrhosis (irrespective of the cause of cirrhosis) among HCC cases (results not shown). 

Among study participants without HBV or HCV infection, we observed a non-significant inverse association with HCC risk for the vegetable-based pattern (OR 0.70, 95% CI 0.49–1.01 for the third tertile compared with the first tertile), and the Western diet pattern was associated with around a two-fold increase in HCC risk in this group (OR 1.97, 95% CI 1.28–3.02 for the third tertile compared with the first tertile). However, no significant association between dietary pattern and HCC risk was found among those with HBV/HCV infection because of the small number of infected controls (data not shown). The test for multiplicative interaction between the Western diet pattern and HBV/HCV infection was significant (Pinteraction = 0.030), and the multiplicative interaction between the Western diet pattern and age was also significant (Pinteraction = 0.038); however, the statistical interactions between the Western diet pattern and sex and between the vegetable-based pattern and HBV/HCV infection, age, and sex were not significant (data not shown). Analysis stratified by age (≥60 years compared with <60 years) suggested a stronger association between HCC and the Western diet pattern in the older group (Table 6). Stratification by sex showed that the preventive effect of the vegetable-based pattern was significant in women, whereas the risk effect of the Western diet pattern was significant in men (Table 6). 

## 4. Discussion

The current study showed that after controlling for potential confounders, a vegetable-based dietary pattern was associated with a reduced HCC risk, whereas a Western diet pattern was associated with an increased risk of HCC. 

Our findings are supported by previous studies reporting an inverse association between HCC and dietary patterns emphasizing high vegetable intake. In two US-based cohort studies [12,13], better adherence to the dietary guidelines for Americans (AHEI-2010) or to the Mediterranean diet was inversely associated with HCC; however, unfortunately, both studies lacked information about HBV and HCV infection status. Both AHEI-2010 [22] and the Mediterranean diet [23] are characterized by high intake of vegetables and fruits and reduced intake of red and processed meats. A combined analysis of two case-control studies in Greece and Italy showed that adherence to a Mediterranean diet was inversely associated with HCC (OR 0.51, 95% CI 0.34–0.75) [24]. In a Shanghai-based cohort study, a vegetable-based dietary pattern identified via PCA was inversely associated with HCC (HR 0.58, 95% CI 0.40–0.84), whereas no association was observed with the fruit- and meat-based patterns [12]. Our findings are also consistent with other diet studies reporting an association between individual food groups and HCC. Similar to our study, increased risk of HCC with red meat intake was reported in the NIH-AARP Diet and Health Study (HR 1.74, 95% CI 1.16–2.61 when comparing the fifth quintile with the first quintile of red meat intake) [25]. 

The biological mechanism by which a vegetable-based dietary pattern could prevent HCC is poorly understood. The protective effect of a dietary pattern could be mediated by the anti-inflammatory and anti-oxidative potential of food components such as fruits, vegetables, and whole grains that are rich in fiber and antioxidants [26]. In general, vegetable-based dietary patterns are associated with a reduced risk of chronic disease, inflammation, and mortality. In the case of HCC, type 2 diabetes mellitus, obesity, and non-alcoholic fatty liver disease are known etiologic factors of the disease. The link between carcinogenesis and dietary patterns could also be related to red and processed meats, a source of carcinogenic N-nitroso compounds, polycyclic aromatic hydrocarbons, and heterocyclic amines produced with curing, smoking, and high-temperature cooking of meat [27].

A limitation of our study is the inherent recall bias in case-control studies, given that cases might recall their diet differently than healthy controls, as well as reverse causation, because patients could alter their dietary habits following disease diagnosis. To address recall bias, we assessed dietary intake the year prior to HCC diagnosis for cases and 1 year prior to recruitment for controls so that measurement error would most likely be non-differential with respect to disease status. Reverse causation was addressed by repeating the analysis among non-cirrhotic cases. HCC is usually preceded by cirrhosis and thus one would assume that patients with cirrhosis would alter their dietary habits. Our findings among non-cirrhotic participants, although not statistically significant, were similar to those in the total study population, minimizing the potential influence of reverse causation. 

Another limitation of the study was some missing BMI information, limiting the use of this measure. However, the demographic characteristics and dietary intake of those with missing BMI data did not differ from those with BMI data. The results of stratification analysis should be interpreted cautiously given the small sample size in the strata. 

The current study used self-reporting to obtain information about several non-dietary risk factors such as education, smoking and alcohol consumption habits, and type 2 diabetes mellitus. We indicated previously that self-reported data about HCC risk factors in this study population were consistent with those obtained from patient records, and thus misreporting is assumed minimal [28]. The reported risk association between HCC and these factors in our case-control study is consistent with previously published results from population-based studies, such as the association between HCC risk and alcohol consumption and cigarette smoking. 

Selection bias could result from recruiting hospital-based cases with advanced-stage HCC; however, selection bias of cases and controls is unlikely in the current study for several reasons. HCC is often detected at late stages [29,30,31]; similarly, around 70% of our cases had advanced-stage HCC. In addition, controls were selected from spouses of patients with various cancers not included in the case definition, because a gastrointestinal, liver, or head and neck cancer diagnosis could be potentially related to HCC and dietary factors. Additionally, spouse controls would most likely share same dietary patterns as the cases, and this could underestimate the effect of dietary patterns on HCC risk. 

PCA has some limitations related to the subjectivity in creation of food groups, selection of factors to retain, and interpretation of these factors. However, we referred to previous literature in forming the food groups used in the analysis and we used scree plots and eigenvalues for factor selection [32,33]. Given the limited research investigating the association between HCC and dietary patterns and food groups, we believe PCA use was justified for exploratory analysis. 

The current study has notable strengths. It is one of the largest case-control studies of HCC among US adults with dietary and non-dietary HCC risk factors, allowing adjustment for a wide range of potential confounders, including education, family history of cancer, BMI, cigarette smoking, alcohol consumption, type 2 diabetes mellitus, and HBV/HCV infection. Previous studies on HCC and diet lacked data on HBV/HCV infection and, therefore, did not adjust for it [12,13,34]. HCC and cirrhosis diagnosis were pathologically confirmed among cases to avoid misdiagnosis of disease. 

In conclusion, we observed an inverse association between a vegetable-based dietary pattern and HCC and a positive association between a Western diet pattern and HCC. These findings emphasize the potential role of dietary intake in HCC prevention. These findings should be confirmed in an ethnically diverse cohort study to assist in developing dietary recommendations to reduce HCC incidence. Moreover, our findings prompt the study of potential HCC prevention strategies among high-risk individuals.

## Figures and Tables

**Table 1:** Food groupings used in the dietary pattern analysis.

Food Group	Food Items
Processed meats	Salami, bologna, sausage, other processed meats, bacon, hot dogs
Red meats	Beef, pork, lamb, hamburger
Organ meats	Liver
Fish	Fish, shell fish
Poultry	Chicken, turkey
Eggs	Eggs
Butter	Butter
Margarine	Margarine
Low-fat dairy	Skim or low-fat milk, yogurt, low fat ice-cream, cottage cheese
High-fat dairy	Whole milk, sour cream, ice cream, cream cheese
Alcohol	Liquor, wine, beer
Tea and coffee	Tea, coffee
Fruits	Grapes, prunes, avocado, bananas, cantaloupe, watermelon, apples, oranges, grapefruit, strawberries, blueberries, peaches, apricots, plums
Fruit juices	Apple juice, orange juice, grapefruit juice, other fruit juice
Cruciferous vegetables	Broccoli, cabbage, cauliflower, brussels sprouts, kale, mustard greens, chard greens
Dark yellow vegetables	Carrots, squash, yams, sweet potatoes
Tomatoes	Tomatoes, tomato juice, tomato sauce
Green leafy vegetables	Spinach, lettuce
Legumes	Beans, peas, lentils, soybeans
Other vegetables	Celery, mushrooms, green pepper, corn, eggplant, summer squash, onion, garlic
Potatoes	Potatoes (baked, boiled, smashed)
French fries	French fries
Whole grains	Oatmeal, dark bread, brown rice, oat bran, wheat germ
Cereal	Cold breakfast cereal
Refined grains	White bread, English muffins, bagels, muffins, white rice, pasta, pancakes, tortillas
Pizza	Pizza
Snacks	Potato chips, crackers, popcorn, pretzels
Nuts	Peanuts, walnuts, other nuts, peanut butter
High-energy drinks	Cola with sugar, other carbonated beverages with sugar
Low-energy drinks	Low-energy cola, other low-energy carbonated beverage
Dressing	Olive oil (as salad dressing or added to bread), oil and vinegar salad dressing
Mayonnaise	Mayonnaise and other creamy salad dressings
Chowder	Chowder or cream soup
Sweets and desserts	Chocolate, candy bars, cookies, brownies, doughnuts, cakes, pies, pastries
Condiments	Sauces, mustard, jam, jelly, syrup, honey, ketchup, artificial sweeteners

**Table 2:** Factor loading matrix for major dietary patterns ^a^.

Food Group	Dietary Pattern	
	Vegetable-Based	Western Diet
Processed meats	---	0.33
Red meats	---	0.41
Organ meats	---	---
Fish	---	0.46
Poultry	---	---
Eggs	---	0.38
Butter	---	---
Margarine	---	---
Low-fat dairy	---	---
High-fat dairy	---	---
Alcohol	---	---
Tea and coffee	---	---
Fruits	0.26	0.20
Fruit juices	0.26	0.33
Cruciferous vegetables	0.51	---
Dark yellow vegetables	0.77	---
Tomatoes	0.66	---
Green leafy vegetables	0.66	---
Legumes	0.76	---
Other vegetables	0.60	---
Potatoes	---	---
French fries	---	---
Whole grains	---	0.60
Cereal	0.27	0.44
Refined grains	---	---
Pizza	---	---
Snacks	---	0.66
Nuts	---	---
High-energy drinks	---	---
Low-energy drinks	---	---
Dressing	0.27	---
Mayonnaise	0.38	---
Chowder	---	---
Sweets and desserts	---	0.72
Condiments	---	---

^a^ For simplicity, only values greater than 0.2 are shown.

**Table 3:** Multivariable adjusted odds ratios (ORs) with 95% confidence interval (CI) for selected demographic factors among cases and controls.

Characteristic	No. (%)		Adjusted OR ^a^ (95% CI)	*p*
	Cases, *n* = 502	Controls, *n* = 671		
Sex				
Female	133 (26.5)	265 (39.5)	1 (reference)	
Male	369 (73.5)	406 (60.5)	1.02 (0.72–1.44)	0.909
Age				
<60 years	177 (35.3)	295 (44.0)	1 (reference)	
≥60 years	325 (64.7)	376 (56.0)	2.57 (1.8–3.66)	<0.0001
Race				
Non-Hispanic White	378 (75.3)	619 (92.3)	1 (reference)	
Other race/ethnicity	124 (24.7)	52 (7.7)	2.65 (1.63–4.30)	<0.0001
Education				
<College education	197 (39.2)	173 (25.8)	1 (reference)	
≥College education	305 (60.8)	498 (74.2)	0.75 (0.54–1.05)	0.096
Alcohol consumption				
None	136 (27.1)	286 (42.6)	1 (reference)	
<60 mL ethanol/day	266 (53.0)	321 (47.8)	1.37 (0.98–1.92)	0.065
≥60 mL ethanol/day	100 (19.9)	64 (9.5)	2.00 (1.23–3.24)	0.005
Cigarette smoking				
No smoking	178 (35.5)	368 (54.8)	1 (reference)	
≤20 pack-years	148 (29.5)	138 (20.6)	1.39 (0.94–2.05)	0.105
>20 pack-years	176 (35.1)	165 (24.6)	1.62 (1.12–2.33)	0.010
Family history of cancer				
No	134 (26.7)	213 (31.7)	1 (reference)	
Yes	368 (73.3)	458 (68.3)	2.06 (1.45–2.94)	0.0001
History of type 2 diabetes mellitus				
None	337 (67.1)	591 (88.1)	1 (reference)	
≤1 year	8 (1.6)	15 (2.2)	1.20 (0.46–3.13)	0.705
>1 year	157 (31.3)	65 (9.7)	3.72 (2.54–5.45)	<0.0001
BMI				
Normal	297 (59.2)	440 (65.6)	1 (reference)	
Overweight	141 (28.1)	192 (28.6)	1.02 (0.71–1.45)	0.924
Obese	64 (12.7)	39 (5.8)	3.18 (1.86–5.43)	<0.0001
HBV/HCV infection				
No	275 (54.8)	658 (98.1)	1 (reference)	
Yes	227 (45.2)	13 (1.9)	53.74 (28.74–100.47)	<0.0001

Abbreviations: OR, odds ratio; CI, confidence interval; BMI, body mass index; HBV, hepatitis B virus; HCV, hepatitis C virus. ^a^ Adjusted were estimated from a multivariable logistic regression model adjusted for sex, age, race, education, alcohol consumption, cigarette smoking, diabetes, BMI, family history of cancer, and HBV/HCV infection. Missing: BMI (n = 134 cases and 330 controls); HBV/HCV infection (n = 4 cases); History of type 2 diabetes mellitus (n = 1 case); Alcohol consumption (n = 1 control).

**Table 4:** General characteristics of controls according to tertile of dietary pattern scores.

Characteristics	Vegetable-Based Pattern			Western Diet Pattern		
	No. (%)		P_trend_	No. (%)		P_trend_
	T1 (Lowest)	T3 (Highest)		T1 (Lowest)	T3 (Highest)	
Sex						
Male	211 (63.4)	176 (52.9)	0.006	171 (51.2)	206 (61.9)	
Female	122 (36.6)	157 (47.2)		163 (48.8)	127 (38.1)	0.005
Age						
≥60 years	161 (48.3)	188 (56.5)		172 (51.5)	182 (54.7)	0.413
<60 years	172 (51.7)	145 (43.5)	0.036	162 (48.5)	151 (45.4)	
Race						
Non-Hispanic White	293 (88.0)	301 (90.4)	0.300	296 (88.6)	299 (89.8)	0.613
Other race	40 (12.0)	32 (9.6)		38 (11.4)	34 (10.2)	
Cigarette smoking						
No smoking	170 (50.9)	181 (54.0)	0.323	171 (51.2)	182 (54.2)	0.713
≤20 pack-years	87 (26.1)	78 (23.3)		85 (25.5)	72 (21.4)	
>20 pack-years	77 (23.1)	76 (22.7)		78 (23.4)	82 (24.4)	
History of type 2 diabetes mellitus						
Yes	28 (8.4)	35 (10.5)	0.371	32 (9.6)	39 (11.7)	0.364
No	305 (91.6)	298 (89.5)		302 (90.4)	294 (88.3)	
Alcohol consumption						
None	139 (41.8)	149 (44.7)	0.856	139 (41.7)	149 (44.7)	0.180
<60 mL ethanol/day	153 (46.1)	152 (45.7)		147 (44.1)	152 (45.7)	
≥60 mL ethanol/day	40 (12.1)	32 (9.6)		47 (14.1)	32 (9.6)	
BMI						
Normal	148 (65.8)	153 (66.8)	0.546	154 (66.7)	140 (62.8)	0.650
Overweight	64 (28.4)	67 (29.3)		62 (26.8)	68 (30.5)	
Obese	13 (5.8)	9 (3.9)		15 (6.5)	15 (6.7)	
HBV/HCV infection						
Yes	4 (1.2)	13 (3.9)		5 (1.5)	13 (3.9)	0.040
No	330 (98.8)	322 (96.1)	0.021	329 (98.5)	323 (96.1)	
Multivitamin use						
Yes	159 (47.8)	200 (60.1)		170 (51.4)	188 (56.1)	0.219
No	174 (52.3)	133 (39.9)	0.001	161 (48.6)	147 (43.9)	

Abbreviations: BMI, body mass index; HBV, hepatitis B virus; HCV, hepatitis C virus.

**Table 5:** Multivariable adjusted ORs and 95% CI for hepatocellular carcinoma according to dietary pattern tertile.

Tertile of Dietary Pattern	Cases	Controls	Adjusted OR (95% CI)	*p*
Vegetable-based pattern				
T1	208	224	1 ^a^ (reference)	
T2	123	218	0.57 (0.40–0.83)	0.003
T3	171	229	0.66 (0.46–0.94)	0.022
P_trend_				0.018
Western diet pattern ^c^				
T1	159	229	1 ^b^ (reference)	
T2	85	218	0.69 (0.45–1.05)	0.086
T3	243	222	1.79 (1.19–2.69)	0.005
P_trend_				0.012

Abbreviations: OR, odds ratio; CI, confidence interval; ^a^ OR adjusted for sex, age, race, history of type 2 diabetes mellitus, body mass index, and hepatitis B or C virus infection; ^b^ OR adjusted for sex, age, alcohol consumption, history of type 2 diabetes mellitus, body mass index, multivitamin use, total calorie intake, and hepatitis B or C virus infection; ^c^ 15 cases and 2 controls were missing multivitamin use.

**Table 6:** Multivariable adjusted ORs and 95% CI for hepatocellular carcinoma according to dietary pattern tertile stratified by sex and age.

Tertile of Dietary Pattern	Group 1			Group 2		
	Cases/Controls	OR (95% CI)	*p*	Cases/Controls	OR (95% CI)	*p*
Sex	Male			Female		
Vegetable-based pattern						
T1	160/148	1 ^a^ (reference)		48/77	1 (reference)	
T2	83/127	0.51 (0.32–0.82)	0.005	40/91	0.66 (0.36–1.19)	0.165
T3	126/132	0.79 (0.51–1.22)	0.286	45/97	0.48 (0.26–0.87)	0.016
Western diet pattern						
T1	117/131	1 ^b^ (reference)		42/98	1 (reference)	
T2	59/131	0.66 (0.38–1.15)	0.143	26/87	0.78 (0.39–1.59)	0.498
T3	179/143	2.27 (1.32–3.90)	0.003	64/79	1.47 (0.75–2.87)	0.260
Age	≥60 years			<60 years		
Vegetable-based pattern						
T1	126/117	1 ^c^ (reference)		82/108	1 (reference)	
T2	79/128	0.52 (0.33–0.81)	0.004	44/90	0.67 (0.34–1.33)	0.253
T3	120/131	0.68 (0.44–1.05)	0.084	51/98	0.61 (0.31–1.19)	0.146
Western diet pattern						
T1	86/123	1 ^d^ (reference)		73/106	1 (reference)	
T2	58/129	0.84 (0.48–1.29)	0.493	27/89	0.48 (0.22–0.21)	0.084
T3	167/123	2.35 (1.41–3.92)	0.001	76/99	1.19 (0.57–2.47)	0.646

Abbreviations: OR, odds ratio; CI, confidence interval; ^a^ OR adjusted for age, race, history of type 2 diabetes mellitus, body mass index, and hepatitis B or C virus infection; ^b^ OR adjusted for age, alcohol consumption, history of type 2 diabetes mellitus, body mass index, multivitamin use, and hepatitis B or C virus infection; ^c^ OR adjusted for sex, race, history of type 2 diabetes mellitus, body mass index, and hepatitis B or C virus infection; ^d^ OR adjusted for sex, alcohol consumption, history of type 2 diabetes mellitus, body mass index, multivitamin use, total calorie intake and hepatitis B or C virus infection.

## Data Availability

The data that support the findings of this study are available on request from the corresponding author. The data are not publicly available due to privacy or ethical restrictions.
